# The Movement disorder associated with NMDAR antibody-encephalitis is complex and characteristic: an expert video-rating study

**DOI:** 10.1136/jnnp-2018-318584

**Published:** 2018-07-21

**Authors:** James A Varley, Alastair J S Webb, Bettina Balint, Victor S C Fung, Kapil D Sethi, Marina A J Tijssen, Timothy Lynch, Shakeeb S Mohammad, Fiona Britton, Matthew Evans, Yael Hacohen, Jean-Pierre Lin, Nardo Nardocci, Tiziana Granata, Russell C Dale, Ming J Lim, Kailash P Bhatia, Anthony E Lang, Sarosh R Irani

**Affiliations:** 1 Oxford Autoimmune Neurology Group, Nuffield Department of Clinical Neurosciences, University of Oxford, Oxford, UK; 2 Department of Neurology, University Hospital, Heidelberg, Germany; 3 The Movement Disorders Unit, Westmead Hospital, Sydney, New South Wales, Australia; 4 Department of Neurology, Augusta University, Augusta, Georgia; 5 Department of Neurology, University Medical Center Groningen, Groningen, The Netherlands; 6 Centre for Brain Health, The Dublin Neurological Institute at the Mater Misericordiae University Hospital, University College Dublin, Dublin, Ireland; 7 Discipline of Child & Adolescent Health, Westmead Children’s Hospital, The University of Sydney, Sydney, New South Wales, Australia; 8 Children's Neurosciences, Evelina Children's Hospital, King's Health Partners Academic Health Science Centre, London, UK; 9 Department of Paediatric Neuroscience, IRCCS Foundation C. Besta Neurological Institute, Milan, Italy; 10 Sobell Department, Institute of Neurology, UCL NHNN, London, UK; 11 Morton and Gloria Shulman Movement Disorders Clinic, The Edmond J. Safra Program in Parkinson's Disease, Toronto Western Hospital, Toronto, Ontario, Canada

**Keywords:** autoantibody, autoimmune, movement disorders, encephalitis, NMDA receptor

## Introduction

N-methyl-D-aspartate receptor antibody-mediated encephalitis (NMDAR-AbE) is an increasingly recognised and treatable encephalitis, with a predilection for children and young adults.^1 2^ As earlier immunotherapy improves outcomes, timely and accurate recognition of NMDAR-AbE is a major clinical aim.^2^

The characteristic polysymptomatic presentation of NMDAR-AbE includes early neuropsychiatric deficits with seizures, autonomic disturbance, reduced consciousness and a movement disorder (MD).^1 2^ This MD, seen in around 90% of cases, can be the presenting feature, particularly in children,^1^ and is typically hyperkinetic with limb plus orofacial involvement.^1–5^ To date, elegant detailed descriptions exist in a few patients.^4^ However, small series have used highly variable phenomenological descriptions.^1–5^ Therefore, the phenomenology of the associated MD lacks consensus. Its clearer description will facilitate confident recognition and enable earlier immunotherapy administration in NMDAR-AbE.

Expert-rater descriptions remain the gold standard to define phenotypes in movement disorders. In this study, ratings from seven experts across 76 videos were used to better define the MD in NMDAR-AbE.

## Methods

### Subjects

Autoimmune neurology researchers contributed 44 videos from 20 patients who met diagnostic criteria for NMDAR-AbE.^e1^ A PubMed search for ‘anti-NMDAR encephalitis’ and ‘NMDAR-antibody encephalitis’, plus associated references, revealed 32 videos from 14 subjects, in eight papers from 14 subjects.^3^ , ^e2–8^ Clinical features, temporal progression, outcomes and investigation findings were collated from case note reviews by the researchers and from data in published papers. Twenty videos from 18 age-matched and sex-matched (age range 2–41 years, median 12, 50% female) disease controls were selected from the literature (online supplementary data).^3^ ^e9–19^

10.1136/jnnp-2018-318584.supp4 Supplementary data

### Movement disorder classification

Seven experts (KB, VF, AEL, TL, NN, KS and MT) established a consensus glossary of terms (online supplementary data, modified from Mohammed *et al* 3). Subsequently, each expert blindly and independently rated 76 videos from 34 patients with NMDAR-AbE (median two videos per patient, range 1–7) plus 20 disease controls, using glossary-derived terms within a standardised data collection sheet (online supplementary data).

## Results

### Cohort characteristics

**Figure 1:**
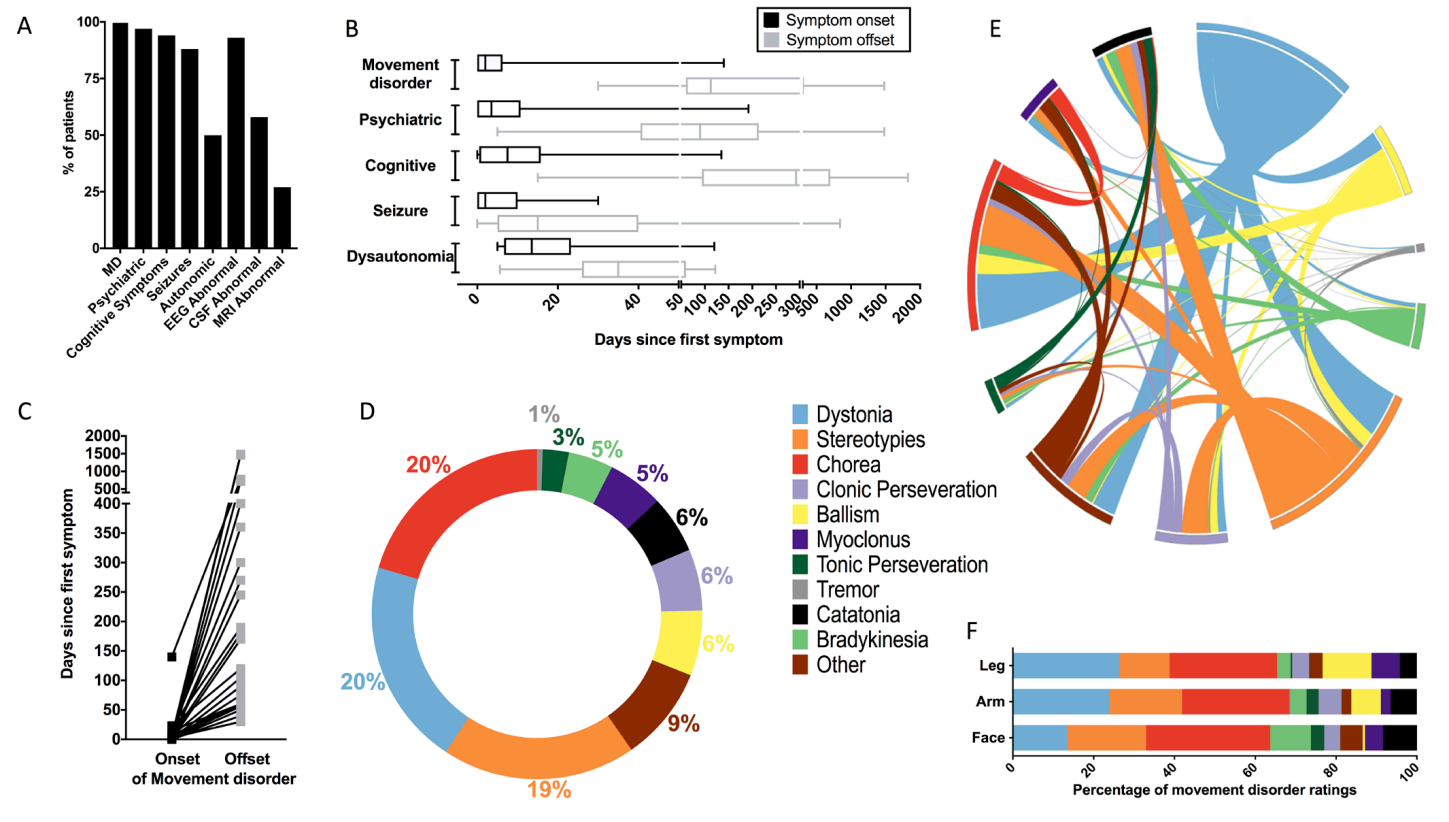
The clinical features and movement disorder evaluations in 34 patients with N-methyl-D-aspartate-antibody encephalitis (NMDAR-AbE). (A) Clinical and investigation findings across the 34 patients whose videos were rated. By definition, all patients had a movement disorder (MD) and other clinical and paraclinical features included psychiatric (n=33/34), cognitive (n=32/34), seizures (n=30/34), autonomic (n=17/34), abnormal electroencephalogram (n=28/30), abnormal cerebrospinal fluid (CSF) (n=19/33) and abnormal MRI (n=9/34). Abnormal CSF findings included any of pleocytosis, oligoclonal bands or raised protein. Two ovarian teratomas were noted in postpubescent women (25 and 32 years). Overall, three cases were adults. (B) Symptom onset and offset in NMDAR-AbE: timings of onset (black) and offset (grey) of the main five symptom categories after first symptom (day 1; median, minimum and maximum values displayed on box and whisker plot), (C) with a particular focus on the timing of the MD in individual patients. Full datasets were available from coauthors and denominators <34 represent variably reported details from the literature-derived videos. Expert classification of phenomenology for 76 videos from patients with NMDAR-AbE (D–F). (D) Dystonia, chorea and stereotypies were the most commonly used terms. For the ‘other’ category, raters used terms including: mutism, stupor, myorhythmia, myokymia, tics, opisthotonus, cerebellar syndrome/ataxia, orofacial dyskinesia, waxy flexibility, oculogyric crises, athetosis, agitation, seizure, startle and vocal perseveration. (E) The interactions between phenomenologies in NMDAR-AbE with co-occurrence of stereotypies, chorea and dystonia shown in a Circos plot,^e20^ based on a co-occurrence matrix within single video ratings. (F) Stereotypies, chorea and dystonia were equally represented in the face, arm and leg, respectively.

From the 34 patients, median age was 7 years (range 0.2–32 years) and 59% were female. Consistent with this young cohort, 6% (2/34) had ovarian teratomas. Peak modified Rankin Scale showed a severe illness (median 5; range 2–5), and, other than the prerequisite MD, the clinical features and investigation findings were in accordance with other series of patients with NMDAR-AbE (figure 1A–B).^1 2^ Overall, the MD was an early feature of the disease (figure 1B) and, in addition to the known prolonged psychiatric symptoms, persisted for a median of 112.5 days (range 30–1482, figure 1C).

### Evaluation of videos

Videos were recorded at a median of 28 days from symptom onset (range 1–690 days). Across all videos, three principle dominant MDs were noted (figure 1D): dystonia (20%), chorea (20%) and stereotypies (19%). Other hyperkinetic phenomenologies included clonic perseveration (6%), ballism (6%), myoclonus (5%), tonic perseveration (3%) and tremor (1%). Hypokinetic terms included catatonia (6%) and bradykinesia (5%). Three representative videos are presented in the online supplementary video 1-3.

Overall, fewer phenomenological terms were used to describe the MDs from disease controls (mean 1.9 per video, range 1–4; online supplementary data) versus patients with NMDAR-AbE (mean 3.4 per video, range 1–7; p<0.0001, Mann-Whitney U-test). This complexity was consistent with the strong inter-rater reliability across the 20 disease control videos in comparison to the 76 NMDAR-AbE videos, for the dominant phenomenology as dystonia (κ=0.65 vs 0.39, controls vs NMDAR-AbE), chorea (κ=0.63 vs 0.39) or stereotypies (κ=0.91 vs 0.22).

Analysis of the frequency of MDs within a single video in the patients with NMDAR-AbE revealed marked coexistence of chorea, dystonia and stereotypies (figure 1E and online supplementary figure 1). Indeed, 97% (33/34) of patients and 79% (60/76) of videos contained two of dystonia, chorea and stereotypies, with 76% (26/34) of patients and 46% (35/76) of videos containing all three phenomenologies. Overall, very similar patterns were observed with inclusion of associated disorders (online supplementary figure 2)^3^ and across ages and gender (data not shown).

### Temporal variability and regional distribution of the movement disorder

To explore these patterns over time, several patients were rated longitudinally. Dystonia, stereotypies and chorea remained prominent but there was marked variability in the MD within an individual patient over the course of a single day and over a number of weeks (median=21 days, range 0–360 days; online supplementary figure 3). Finally, the anatomical distribution of these phenomenologies revealed similar ratings in the face, arm and leg (figure 1F).

## Discussion

NMDAR-AbE is a severe yet treatable neurological disorder in which prompt administration of immunotherapies improves outcomes.^2^ The MD is a common and well-emphasised feature of disease descriptions, yet until now lacked an expert-based gold-standard description.^1–5^ From this study, the MD was an early and persistent feature and, from a large collection of videos, it was characterised by diverse, widely distributed hyperkinetic phenomenologies, with prominent dystonia, chorea and stereotypies, with almost complete absence of tremor and tics and a relative paucity of myoclonus. Furthermore, it was complex to describe using conventional MD terminology, yet the above features may help differentiate this mixed movement disorder from important differential diagnoses in routine clinical practice including primary psychiatric disorders, childhood metabolic disturbances and drug intoxication. Indeed, the coexistence of widely distributed chorea, dystonia and stereotypies is rare in other neurological conditions (expert panel, unpublished observations) and may reflect the targeted autoantibody-mediated downregulation of NMDARs.

In this study, expert-rater feedback stated ‘*our definition and description of hyperkinetic movements do not cover some of these patients* *’*and ‘*reviewing all of these videotapes was a challenge particularly to preconceived and established phenomenological categorisations. Not infrequently I was dissatisfied with the final category that I chose*’. This difficulty in classifying patients with conventional terms is reflected in the inter-rater kappa statistics and the greater variety of terms used to describe patients with NMDAR-AbE. These kappa values could be interpreted as reflecting variability between expert-raters, but they showed good agreement across other varied control disorders and many terms are not interchangeable. However, potential study limitations include the lack of accompanying clinical histories and standardised video assessments, the overall young age and marked disease severity, the surprisingly prolonged duration of the MD and an intrinsic bias towards recording the most unusual movements. Future extension studies could also incorporate a consecutive series of patients with a more exhaustive list of control mixed movement disorders.

In summary, this clinical constellation of phenomenologies, with the unusual triad of dystonia, stereotypies and chorea, should, in the correct clinical context, provide clinicians with greater confidence in diagnosing this common cause of encephalitis, allowing earlier immunotherapy administration and improved patient outcomes.

10.1136/jnnp-2018-318584.supp1 Supplementary video

10.1136/jnnp-2018-318584.supp2 Supplementary video

10.1136/jnnp-2018-318584.supp3 Supplementary video
