# Supplementary material for: The Movement disorder associated with NMDAR antibody-encephalitis is complex and characteristic: an expert video-rating study
Source: J Neurol Neurosurg Psychiatry. 2018 Jul 21;90(6):724–6. doi: 10.1136/jnnp-2018-318584 (PMC6581096; doi:10.1136/jnnp-2018-318584)
Supplement: Supplementary data [file jnnp-2018-318584supp004.pdf]

Supplementary Material

eReferences

Graus criteria for NMDAR-AbE[1]
Literature patient videos[2-8]
Control patient videos[9-19]

Chord Diagram
Plotted using Circos.[20]

Glossary (Modified from Mohammad et al – ref 3 in main manuscript)

Tremor: The consensus statement of the movement disorder society on tremor defines it as “Tremor is a rhythmic back-and-forth or oscillating involuntary movement about a joint axis.”[21]

> **Action tremor:** Action tremor is any tremor that is produced by voluntary contraction of muscle, including postural, isometric, and kinetic tremor. The latter includes intention tremor (also known as terminal kinetic tremor).[21]
> **Dystonic tremor:** A spontaneous oscillatory, rhythmical, although often inconstant, patterned movement produced by contractions of dystonic muscles often exacerbated by an attempt to maintain primary (normal) posture. The dystonic tremor may not be relieved by allowing the abnormal dystonic posture to fully develop without resistance (“null point”). Dystonic tremor may be difficult to distinguish from other types of tremor.[22]
> **Rest tremor:** Rest tremor is defined by tremor that occurs in a body part that is not voluntarily activated and is completely supported against gravity.[21]

Athetosis: Athetosis is a slow, continuous, involuntary writhing movement that prevents maintenance of a stable posture. In contrast to chorea, in athetosis the same regions of the body are repeatedly involved.[23]

Ballism: Repetitive, but constantly varying, large amplitude involuntary movements of the proximal parts of the limbs. This activity is almost ceaseless and movements are often complex and combined.[24]

Chorea: The ad hoc Committee on Classification of the World Federation of Neurology has defined chorea as "a state of excessive, spontaneous movements, irregularly timed, non-repetitive, randomly distributed and abrupt in character. These movements may vary in severity from restlessness with mild intermittent exaggeration of gesture and expression, fidgeting movements of the hands, unstable dance-like gait to a continuous flow of disabling, movements with a continuum into ballism, where ballism is a more proximal form of chorea.[25]

Dystonia: A Consensus Committee was established under the auspices of the Dystonia Medical Research Foundation, the Dystonia Coalition, and the European Dystonia Cooperation in Science and Technology (COST) Action which proposed the following definition of Dystonia: “Dystonia is a movement disorder characterized by sustained or intermittent muscle contractions causing abnormal, often repetitive, movements, postures, or both. Dystonic movements are typically patterned, twisting,

and may be tremulous. Dystonia is often initiated or worsened by voluntary action and associated with overflow muscle activation."[22]

Myoclonus: Myoclonus is a sequence of repeated, often non-rhythmic, brief shock-like jerks due to sudden involuntary contraction (positive myoclonus) or relaxation (negative myoclonus or asterixis) of one or more muscles.[23]

Tics: Tics are repeated, individually recognizable, intermittent movements or movement fragments that are almost always briefly suppressible and are usually associated with awareness of an urge to perform the movement.[23]

Stereotypy: Stereotypy has been defined variably over the years. The latest proposed definition from Edwards et.al defines stereotypy as "a non–goal-directed movement pattern that is repeated continuously for a period of time in the same form and on multiple occasions, and which is typically distractible"[26.] However, a widely accepted definition from Jankovic defines stereotypy as: "involuntary or unvoluntary, coordinated, patterned, repetitive, rhythmic, purposeless but seemingly purposeful or ritualistic movement, posture or utterance."[27]

Myorhythmia: Is defined as a repetitive, rhythmic, frequently jerky movement with a slow (1-4Hz) frequency, primarily affecting cranial and limb muscles. It typically occurs at rest but can occur with maintenance of posture and voluntary movements, disappearing with sleep.[28]

Akathisia: Akathisia refers to a subjective feeling of inner restlessness, prompting movement in order to reduce to relieve this feeling. Motor corollary are often complex and repetitive and can involve almost any part of the body.

Myokymia: Myokymia is characterised by persistent undulating muscle quivering associated with non-rhythmic, irregular bursts of normal motor unit potentials usually occurring in doublets or triplets, with a broad range (5-150 Hz) of frequencies. Myokymia most often involves the eyelids but can affect other facial muscles.[29]

Opsoclonus: Opsoclonus can be defined as the intrusion into fixation of involuntary, repetitive, rapid, multi-vectorial, conjugate ocular saccades that are irregular in amplitude and frequency and occur in all directions without intersaccadic interval.[30]

Perseveration: It can be defined as the inappropriate persistence of word, thought, or action in response to a given stimulus. *Motor perseveration* is the inability to stop a movement or a series of movements after the task is complete. In recurrent motor perseveration, the patient persistently returns to a previously completed motor program, and in afferent or continuous motor perseveration, the patient cannot end a recently completed motor program.[31]

> **Clonic perseveration:** In clonic perseveration a performance, once initiated, is repeated indefinitely without interruption; for example, the patient may continuously repeat letters when writing a word or continue to draw circles though asked to draw only one.[32]
>
> **Intentional perseveration:** In intentional perseveration a performance is repeated when, and only when, a new performance is intended. An example,

cited by Liepmann, is the patient who has blown out a flame and in the subsequent performance of different duties repeats the blowing movement. **Tonic perseveration:** Tonic perseveration refers to persistence of a muscle contraction once initiated in the absence of an ongoing cue. An example, cited by Liepmann is the patient who having grasped an object cannot voluntarily relax the hand although the effort is made to do so.[33]

Bradykinesia/Akinesia: Bradykinesia is characterized by slowness of movement and has been linked to Parkinson's disease and other disorders of the basal ganglia. Rather than being a slowness in initiation (akinesia), bradykinesia describes a slowness in the execution of movement and a decrease in its amplitude.
Akinesia is the inability to initiate movement due to difficulty selecting and/or activating motor programs in the central nervous system.
We use the term akinesia as an umbrella term similar to previous usage[34] to include bradykinesia (slowness of movement) and hypokinesia (poverty of movement, and movements that are smaller than intended), and the progressive fatiguing and decrement of repetitive alternating movements seen during finger or foot tapping[4].

Catatonia: This has recently been redefined in DSM-5[35] as the presence of three or more of:

- Catalepsy – passive induction of a posture held against gravity
- Waxy flexibility – slight and even resistance to positioning by examiner
- Stupor – no psychomotor activity; not actively relating to environment
- Agitation – not influenced by external stimuli
- Mutism – no, or very little verbal response – NB not applicable if established aphasia
- Negativism – opposing or not responding to instructions or external stimuli
- Posturing - spontaneous and active maintenance of a posture against gravity
- Mannerisms – odd caricature of normal actions
- Stereotypies – repetitive, abnormally frequent, non-goal directed movements
- Grimacing
- Echolalia/Echopraxia – mimicking another’s speech/movements respectively

Data Collection Sheet

	video 1		video 2		video 3		video 4	
	dominant (D) or associated (A)?*	Distribution	dominant (D) or associated (A)?*	Distribution	dominant (D) or associated (A)?*	Distribution	dominant (D) or associated (A)?*	Distribu
dystonia								
chorea								
myoclonus								
clonic perseveration								
ballism								
tonic perseveration								
tremor								
stereotypies								
bradykinesia / akinesia								
other - please specify								
None								

Disease controls

These included both pure MDs with chorea (Sydenham's, n=3), stereotypies (Rett's syndrome n=2; Autism, n=1) and dystonia (DYT1, n=2; DYT16, n=2), and complex mixed MDs (ADCY5, n=3; FOXG1, n=3; Ataxia Telangiectasia, n=1; Myoclonus Dystonia, n=2; Neuroacanthocytosis, n=1).

Statistical Analysis

Statistics included inter-rater reliability (Fleiss-kappa) and Mann-Whitney U test. Analyses were performed in GraphPad Prism v7 and Microsoft Excel.

VIDEO LEGENDS

A selection of representative videos from patients with age and percentage (number) of dominant movement disorders rated by the experts.

Supplementary Video 1. Age 7 years, 75% (6/8) chorea, 12.5% (1/8) 'other' and 12.5% (1/8) dystonia.

Supplementary Video 2. Age 11 years, 71.4% (5/7) stereotypies, 14.3% (1/7) ballism and 14.3% (1/7) catatonia.

Supplementary Video 3. Age 4 years, 44.4% (4/9) chorea and 33.3% (3/9) ballism, 11.1% (1/9) dystonia and 11.1% (1/9) stereotypies.

Supplementary Figure 1. Co-occurrence of movement disorder phenomenology by dominant movement disorder

The most common MD rated within a video was used to compare co-occurrence of other phenomenologies within that video, for dystonia (A), stereotypies (B) and chorea (C).

Supplementary Figure 2. Phenomenological descriptions of videos for dominant plus associated disorders.

Overall, the patterns and proportions are strikingly similar to the dominant disorders alone. Slightly lower overall kappa values were noted, likely due to the sensitivity of Fleiss-kappa values to an increased number of responses from multiple raters: dystonia (κ= 0.44 vs 0.46, controls vs NMDAR-AbE), chorea (κ=0.48 vs 0.34), and stereotypy (κ= 0.74 vs 0.17). (A) The summarised data for expert-ratings for 76 videos from patients with NMDAR-antibody encephalitis. For other, raters used terms including mutism, stupor, myorhythmia, myokymia, tics, opisthotonus, cerebellar syndrome/ataxia, orofacial dyskinesia, waxy flexibility, oculogyric crises, athetosis, agitation, seizure, startle and vocal perseveration. (B) Striking co-occurrence of stereotypies, chorea and dystonia shown in a Circos[20] plot, based on a co-occurrence matrix within single video ratings.

Supplementary Figure 3. Description of movement disorder over time in patients with multiple videos.

The movement disorder is highly variable over minutes and hours as well as over days to weeks. These figures show the dominant movement disorder calls made by each expert where more than one video was available for a patient. Each bar displays the different movement disorders called and gaps between clusters of bar allow distinction between groups of videos from a single patient. (A) Sequential videos of the same patient, filmed on the same day. (B) Videos of the same patient, filmed over a range of timepoints (median= 21 days, range 0-360 days).

Bibliography

1. Graus F, Titulaer MJ, Balu R, et al. A clinical approach to diagnosis of autoimmune encephalitis. *Lancet Neurol* 2016;15:391-404.

2. Armangue T, Leypoldt F, Malaga I, et al. Herpes simplex virus encephalitis is a trigger of brain autoimmunity. *Ann Neurol* 2014;75:317-323.

3. Baizabal-Carvallo JF, Stocco A, Muscal E, Jankovic J. The spectrum of movement disorders in children with anti-NMDA receptor encephalitis. *Mov Disord* 2013;28:543-547.

4. Bayreuther C, Bourg V, Dellamonica J, Borg M, Bernardin G, Thomas P. Complex partial status epilepticus revealing anti-NMDA receptor encephalitis. *Epileptic Disord* 2009;11:261-265.

5. McCarthy A, Dineen J, McKenna P, et al. Anti-NMDA receptor encephalitis with associated catatonia during pregnancy. *J Neurol* 2012;259:2632-2635.

6. Poloni C, Korff CM, Ricotti V, et al. Severe childhood encephalopathy with dyskinesia and prolonged cognitive disturbances: evidence for anti-N-methyl-D-aspartate receptor encephalitis. *Dev Med Child Neurol* 2010;52:e78-82.

7. Wong-Kisiel LC, Ji T, Renaud DL, et al. Response to immunotherapy in a 20-month-old boy with anti-NMDA receptor encephalitis. *Neurology* 2010;74:1550-1551.

8. Xia C, Dubeau F. Teaching Video NeuroImages: dystonic posturing in anti-NMDA receptor encephalitis. *Neurology* 2011;76:e80.

9. Fasano A, Nardocci N, Elia AE, Zorzi G, Bentivoglio AR, Albanese A. Non-DYT1 early-onset primary torsion dystonia: comparison with DYT1 phenotype and review of the literature. *Mov Disord* 2006;21:1411-1418.

10. Goto S, Yamada K, Shimazu H, et al. Impact of bilateral pallidal stimulation on DYT1-generalized dystonia in Japanese patients. *Mov Disord* 2006;21:1785-1787.

11. Guehl D, Cuny E, Tison F, et al. Deep brain pallidal stimulation for movement disorders in neuroacanthocytosis. *Neurology* 2007;68:160-161.

12. Cimaz R, Gana S, Braccesi G, Guerrini R. Sydenham's chorea in a girl with juvenile idiopathic arthritis treated with anti-TNFalpha therapy. *Mov Disord* 2010;25:511-514.

13. Camargos S, Lees AJ, Singleton A, Cardoso F. DYT16: the original cases. *J Neurol Neurosurg Psychiatry* 2012;83:1012-1014.

14. Goldman S, Temudo T. Hand stereotypies distinguish Rett syndrome from autism disorder. *Mov Disord* 2012;27:1060-1062.

15. Kobylecki C, Damodaran D, Kerr B, Newton RW, Silverdale MA. Prominent Lower-Limb Involvement in a Family with Myoclonus-Dystonia. *Movement Disorders Clinical Practice* 2014;1:115-117.

16. Myers PJ, Kane KE, Porter BG, Mazzaccaro RJ. Sydenham Chorea: rare consequence of rheumatic fever. *West J Emerg Med* 2014;15:840.

17. Thompson S, Iyer A, Byrd P, Taylor M, Spinty S. Dopa-Responsive Dystonia and Chorea as a Presenting Feature in Ataxia-Telangiectasia. *Movement Disorders Clinical Practice* 2014;1:249-251.

18. Carapito R, Paul N, Untrau M, et al. A de novo ADCY5 mutation causes early-onset autosomal dominant chorea and dystonia. *Mov Disord* 2015;30:423-427.

19. Cellini E, Vignoli A, Pisano T, et al. The hyperkinetic movement disorder of FOXG1-related epileptic-dyskinetic encephalopathy. *Dev Med Child Neurol* 2016;58:93-97.

20. Krzywinski M, Schein J, Birol I, et al. Circos: an information aesthetic for comparative genomics. *Genome Res* 2009;19:1639-1645.

21. Deuschl G, Bain P, Brin M. Consensus statement of the Movement Disorder Society on Tremor. Ad Hoc Scientific Committee. *Mov Disord* 1998;13 Suppl 3:2-23.

22. Albanese A, Bhatia K, Bressman SB, et al. Phenomenology and classification of dystonia: a consensus update. *Mov Disord* 2013;28:863-873.

23. Sanger TD, Chen D, Fehlings DL, et al. Definition and classification of hyperkinetic movements in childhood. *Mov Disord* 2010;25:1538-1549.

24. Meyers R. Ballismus. In: Vinken PJ BG, editors. Handbook of clinical neurology. North-Holland, Amsterdam1968.

25. Barbeau A, Duvoisin RC, Gerstenbrand F, Lakke JP, Marsden CD, Stern G. Classification of extrapyramidal disorders. Proposal for an international classification and glossary of terms. *J Neurol Sci* 1981;51:311-327.

26. Edwards MJ, Lang AE, Bhatia KP. Stereotypies: a critical appraisal and suggestion of a clinically useful definition. *Mov Disord* 2012;27:179-185.

27. Jankovic J. Stereotypies. In: Marsden CD FS, eds. Movement Disorders, 3rd ed, 1994.

28. Baizabal-Carvallo JF, Cardoso F, Jankovic J. Myorhythmia: phenomenology, etiology, and treatment. *Mov Disord* 2015;30:171-179.

29. Yaltho TC, Jankovic J. The many faces of hemifacial spasm: differential diagnosis of unilateral facial spasms. *Mov Disord* 2011;26:1582-1592.

30. Averbuch-Heller L, Remler B. Opsoclonus. *Semin Neurol* 1996;16:21-26.

31. Daroff RB MJ. Neurology in Clinical Practice: Elsevier Health Sciences, 2012.

32. Liepmann H. Die perseveration. In: Liepmann H e. Ueber Storungen des Handelns bei Gerhirnkranken. Berlin: Karger, 1905.

33. Fung VS, Morris JG, Leicester J, Soo YS, Davies L. Clonic perseveration following thalamofrontal disconnection: a distinctive movement disorder. *Mov Disord* 1997;12:378-385.

34. Abdo WF, van de Warrenburg BP, Burn DJ, Quinn NP, Bloem BR. The clinical approach to movement disorders. *Nat Rev Neurol* 2010;6:29-37.

35. Tandon R, Heckers S, Bustillo J, et al. Catatonia in DSM-5. *Schizophr Res* 2013;150:26-30.

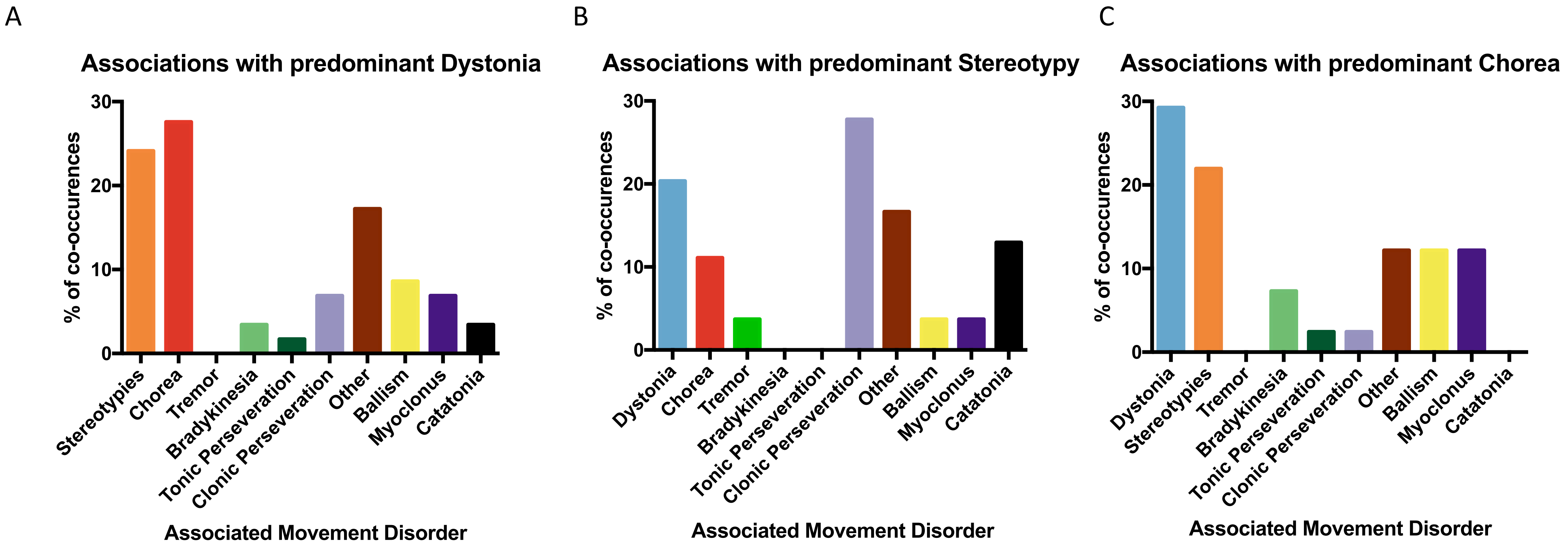

Supplementary Figure 1

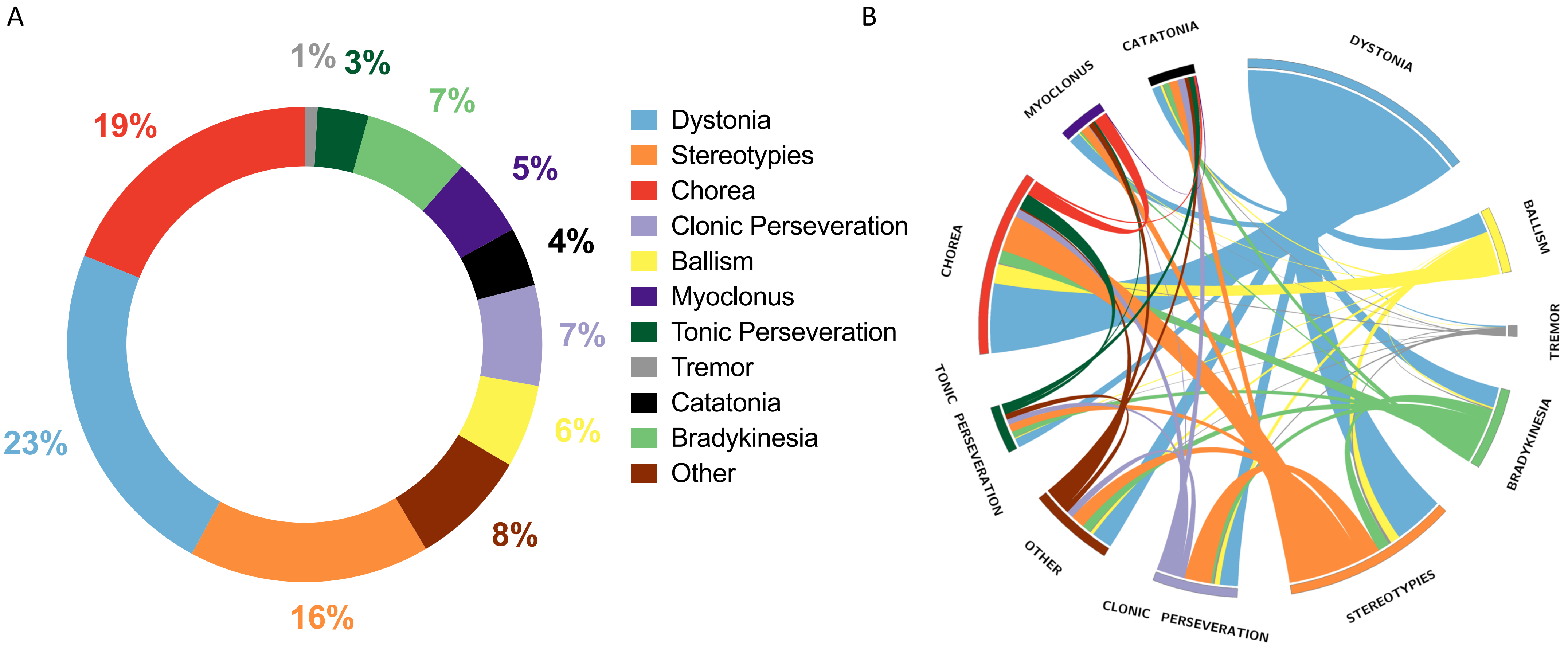

Supplementary Figure 2

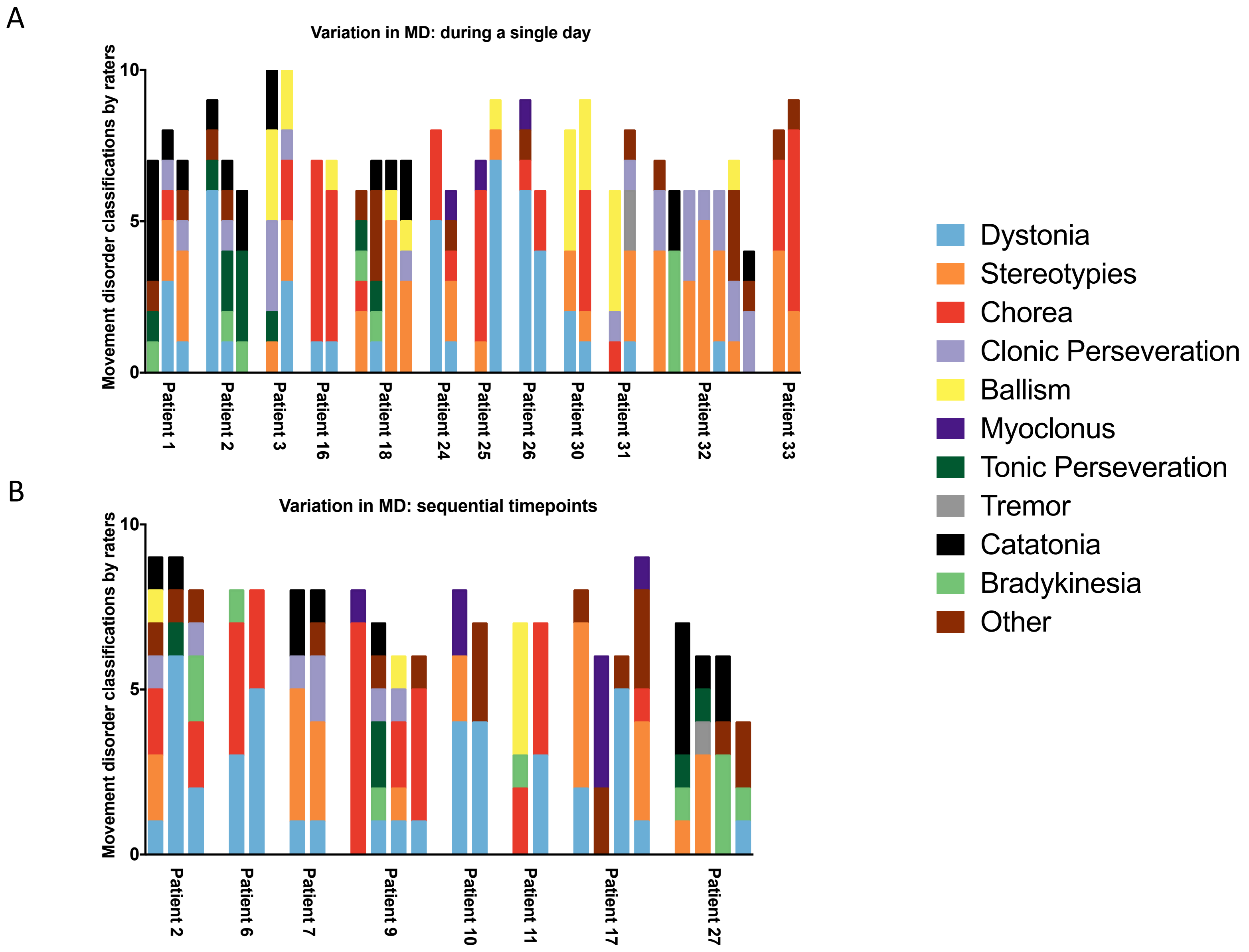

Supplementary Figure 3
